# Development of Fit-for-Purpose, High Quality Proficiency Samples for Interlaboratory Evaluation of RT-PCR Detection of HPAI H5N1 in Milk

**DOI:** 10.1007/s12560-026-09699-x

**Published:** 2026-06-08

**Authors:** Emily L. Smith, Yuhan Jin, Megan R. Miller, Kirstin Frost, Jodie Ulaszek, Sarah M. Nemser, Laura B. Goodman, Steffen Uhlig, Anja Schlierf, Karina Hettwer, Matthew Kmet, Andriy Tkachenko, Olgica Ceric, Gregory H. Tyson, Ravinder Reddy

**Affiliations:** 1 Center for Veterinary Medicine, U.S. Food and Drug Administration, 8401 Muirkirk Road, Laurel, MD 20708 USA; 2 Human Foods Program, U.S. Food and Drug Administration, 6502 South Archer Road, Bedford Park, IL 60501 USA; 3 QuoData – Quality & Statistics, Prellerstraße 14, 01309 Dresden, Germany; 4 Institute for Animal Health, Cornell University, 235 Hungerford Hill Rd, Ithaca, NY 14853 USA; 5 Institute for Food Safety and Health, Illinois Institute of Technology, 6502 South Archer Road Bedford Park, Chicago, IL 60501 USA

**Keywords:** HPAI, H5N1, Milk, Real-time PCR, Digital PCR, Proficiency

## Abstract

The increased demand for highly pathogenic avian influenza (HPAI) testing in milk highlights the critical need for well-characterized samples that enable meaningful evaluation of current detection methods. In this study, we focus on the development and rigorous characterization of artificially contaminated spiked milk samples, forming the foundation for reliable assessment of assay performance. By using reverse transcriptase digital PCR (RT-dPCR), we obtained precise genome copy (GC) measurements of our virus stock and of spiked samples, producing accurate quantification even at low viral loads. In parallel, the U.S. Department of Agriculture National Veterinary Services Laboratories’ (USDA-NVSL’s) influenza A matrix gene (IAV M) real-time reverse transcriptase (rRT-PCR) assay was employed as the main detection assay, serving as a tool to validate the sample preparation methodology. The goal of this work is to show that robust, reproducible, and analytically traceable samples can be generated to support HPAI method evaluation in proficiency exercises (PEs) distributed nationwide. Low between-sample standard deviations, ranging from 0 to 0.10 rRT-PCR Ct values for replicate samples, fall below the theoretical threshold indicative of heterogeneity, along with RT-dPCR determinations of sample GCs affirm the homogeneity of the developed samples. Parallel analysis of corresponding milk-based and phosphate buffered saline (PBS)-based samples showed no indication of a matrix effect. No significant shifts of Ct value or of measured GCs were observed over time, proving samples to be stable for at least 28 days when stored at -80 °C. The defined assay sensitivity, expressed as the level of detection 50% (LOD_50%_), of the USDA-NVSL’s IAV M rRT-PCR assay was determined to be 3.2 GCs/rRT-PCR reaction for milk samples and 5.5 GCs/rRT-PCR reaction for PBS samples, but this difference is not significant. This work sets the basis for determining laboratory competency, providing confidence in HPAI testing results for ongoing and future surveillance.

## Introduction

In March 2024, the first confirmed case of highly pathogenic avian influenza A H5N1 (HPAI) infection in dairy cattle was reported in Texas (Burrough et al., 2024). Raw milk has been shown to contain high concentrations of infectious H5N1 virus, raising significant public health concerns regarding the safety of milk supply (Caserta et al., 2024; Mostafa et al., 2024). Early detection of HPAI virus in bulk tank milk is critical, as it enables timely implementation of containment measures (Stenkamp-Stram et al., 2025). In response, the USDA Animal and Plant Health Inspection Service (APHIS) issued federal orders requiring testing and reporting of influenza A in dairy cattle prior to interstate movement and, later, testing of raw milk under the National Milk Testing Strategy (NMTS) (USDA, April 2024; USDA, November 2025). These measures have driven the rapid expansion of HPAI testing in dairy cattle and milk across veterinary and public health laboratory networks.

Much of the routine testing for HPAI H5N1 in milk relies on rRT-PCR assays developed and recommended by the USDA National Veterinary Services Laboratories (NVSL). NVSL’s standard procedure for rRT-PCR Detection of Influenza A and Avian Paramyxovirus Type-1 (NVSL-SOP-0068) is listed among the approved National Animal Health Laboratory Network (NAHLN) documents (USDA, December 2025). The document includes several different assays, along with extraction kit and RT-PCR kit options and no specific matrix-related recommendations. Specific considerations for handling milk samples were later supplemented by the introduction of NVSL-WI-1843 in September 2024 (USDA, December 2025). In this study, we specifically focus on the rRT-PCR protocol for detection of the IAV M gene, the typical initial screening assay performed prior to more specific subtyping assays, while also following the recommendations of NVSL-WI-1843. NVSL-SOP-0068 and NVSL-WI-1843 are not publicly available and are typically only provided to NAHLN approved laboratories who perform HPAI testing.

Reliable analytical data relies on both a properly validated method and appropriate implementation with in-house laboratory verification; reliability of a method within individual laboratories can be confirmed via participation in PEs, such as a proficiency test (PT) or interlaboratory comparison exercise (ICE), where PTs are graded exercises and ICEs are more exploratory exercises, allowing laboratories to evaluate their methodology without pass/fail criteria. To identify performance gaps in current methodologies and ensure that such testing remains reliable and comparable across laboratories, there is a clear need to prepare a structured PE focused specifically on HPAI detection in milk. The collaborative PEs offered by the FDA’s Veterinary Information Laboratory Response Network (Vet-LIRN) and Moffett Proficiency Testing Laboratory (MPTL), including exercises for another zoonotic virus, SARS-CoV2, show that they are well positioned to prepare such material for nationwide PEs (Nemser et al., 2021; Deng et al., 2023; Singh et al., 2025).

The significance and impact of a PE relies on the relevance of the chosen matrix, the robustness and traceability of the test samples, and the assay used to characterize those samples. Without knowing the limitations of a detection assay, you cannot effectively challenge participants, and you cannot define the limitations of an assay without well-prepared, low-level samples. The sensitivity and specificity requirements of rRT-PCR detection also insist upon rigorous sample characterization. The characterization of the samples in this study—particularly their suitability for sensitivity assessment was performed in accordance with the relevant standards, including ISO 16140-4:^2020^ and ISO/TS 27878:^2023^ for determining the LOD, as well as ISO 13528:^2022^ for assessing sample homogeneity and stability (HG/SB). Although these standards provide the general framework, the analysis applied in this study extends beyond their requirements by incorporating innovative, more comprehensive analytical procedures, 

following a QuoData certified approach 

(QuoData GmbH, n.d;

Uhlig et al., 2015; Uhlig et al., 2018).

Here we discuss the production of high-quality proficiency samples, using the NVSL’s rRT-PCR IAV M assay for the detection of HPAI in milk as an internal sample characterization assay for MPTL. Using inactivated HPAI A (H5N1) spiked into milk we evaluated: the duration of sample stability, sample homogeneity across spike levels, method accuracy as agreement with RT-dPCR derived absolute GC assessment, matrix effect, and method sensitivity using probability of detection (POD) GC levels in milk versus in phosphate buffered saline (PBS) to determine the LOD_50%_. This study demonstrates that the sample preparation and testing approaches used in the MPTL laboratory are suitable for producing milk-based proficiency samples of suitable quality with high degree of confidence.

## Materials/Methods

### Virus Stock and Controls

Binary ethylenimine (BEI) inactivated H5N1 virus (138-ADV I 2401; NVSL-FM-1420.08), a positive extraction control (PEC) (206-ADV I 2401), negative extraction control (NEC) (209-ADV 1 2301), and positive amplification control (PAC) (203 ADV I 0704) were provided by USDA-APHIS-NVSL (Ames, IA). Upon arrival, virus stocks were stored at −80 °C until use. The H5N1 virus stock was determined to be 4.35 × 10^5^ GCs/µL, as described in the RT-dPCR section below. Additional controls included the Xeno Internal Positive Control RNA (IPC), which is included in each sample extraction to confirm consistent extraction efficiency and to check for PCR inhibition, and nuclease-free water (Thermo Fisher Scientific, Waltham, MA) as a no template control (NTC). Controls were prepared and stored according to guidance from the provided reagent data sheets and NVSL-SOP-0068.05.

### Milk Matrix

A half-gallon of pasteurized, store bought whole milk was purchased to be used to simulate HPAI-contaminated milk samples (Chicago, IL). Milk was stored at 4 °C and screened for IAV M prior to use for samples. The Xeno IPC was included in the screening process to ensure that the milk showed no inhibitory effects on rRT-PCR. After screening, the volume of milk needed to make the required number of replicates samples, plus overage to account for pipetting error, was aliquoted into Lo-Bind tubes (Eppendorf SE, Hamburg, Germany) to be bulk inoculated with inactive H5N1 virus.

### Sample Spiking

GC targets were based on endpoint rRT-PCR input; thus, back calculation was used to calculate spiking targets in pre-extraction milk samples. The back calculation, shown below, accounted for all steps of the protocol: dilution of sample prior to extraction (DF = dilution factor), extraction volume, elution volume, and volume of RNA template added to the rRT-PCR reaction.

$$ \begin{aligned} \begin{array}{*{20}c} {{\mathrm{target~GCs}}/\mu {\mathrm{L}}} \\ {{\mathrm{in~spiked~milk~sample}}} \\ \end{array} {\mathrm{~}} \cong {\mathrm{~}} & {\mathrm{~}}\frac{{{\text{target~in~rRT - PCR~reaction~}}\left( {{\mathrm{GCs}}} \right)}}{{{\text{rRT - PCR~reaction~volume~}}\left( {\mu {\mathrm{L}}} \right)}}{\mathrm{~~}} \\ & {\mathrm{*~~}}\frac{{{\mathrm{RNA~elution~volume~}}\left( {\mu {\mathrm{L}}} \right)}}{{{\mathrm{extraction~input~volume~}}\left( {\mu {\mathrm{L}}} \right)}}{\mathrm{~~*~}}\begin{array}{*{20}c} {{\mathrm{DF~of~sample~}}} \\ {{\text{pre - extraction}}} \\ \end{array} \\ \end{aligned} $$

Once GC targets of the milk samples were calculated, a dilution scheme of the previously quantified virus stock was created. Following a tenfold dilution scheme, the virus stock was serially diluted in nucleic acid dilution solution (NADS) (supplied with the Xeno IPC RNA, Thermo Fisher Scientific) out to the 1/10,000 (10^− 4^). The final dilution, or spike, to obtain the desired GCs was performed directly in the bulk milk sample by adding a small volume (~ 50–100 µL) of the closest tenfold dilution to the calculated volume of milk (~ 1–3 mL, depending on required sample replicates).

Throughout the process, all reagents, milk, samples, and virus stock/dilutions were kept on ice unless being directly handled. After spiking, the bulk sample was briefly homogenized by pulse vortexing at 1400–1500 rpm for ~ 5–10 s. To pull any sample volume from the sides and lid of the tube, the sample was briefly spun down in a mini centrifuge and then aliquoted into Lo-Bind tubes as 100 µL samples. Day 0 samples were extracted immediately following spiking and the remaining samples were stored at −80 °C until timepoint testing.

### Extraction

Samples were extracted following NVSL-SOP-0068.05 and NVSL-WI-1843. Sample extraction was automated on the Kingfisher Flex instrument (Thermo Fisher Scientific) following the simple workflow in Thermo Fisher’s MagMAX CORE User Guide (Chap. 3).

Samples, samples dilutions, controls, and carrier RNA was kept on ice for the duration of extraction setup. Prior to extraction, samples were diluted 1/3 in PrimeStore Molecular Transport Medium (MTM) (Longhorn Vaccine and Diagnostics, Bethesda, MD) by adding 200 µL of MTM to each 100 µL sample. 200 µL of each diluted milk sample was added to the extraction plate. Two µL of Xeno IPC RNA and 2 µL carrier RNA (1 µg/µL, Thermo Fisher Scientific, Waltham, MA) was added to the lysis/binding mix just prior to distribution into sample wells. A PEC sample and a NEC sample was included in each extraction. The volume (µL) of eluted RNA from samples was recorded as they were pipetted into labelled Lo-Bind Tubes. Tubes were placed on ice until all samples were removed from the elution plate and then stored at −20 °C until rRT-PCR analysis.

### rRT-PCR

The rRT-PCR assay was set up according to NVSL-SOP-0068.05 on the Applied Biosystems™ 7500 Fast (ABI7500 Fast) using version 2.3 software (Thermo Fisher Scientific, Waltham, MA). The extracted RNA from samples was reverse transcribed to cDNA to be amplified with master mix created by combining primers and a probe targeting the IAV matrix gene (IAV M) (Integrated DNA Technologies, IDT, Coralville, IA) the Xeno IPC VIC assay (Thermo Fisher Scientific, Waltham, MA), and the AgPath-ID One-Step RT-PCR kit reagents. The primers and probe sequences for IAV M are confidential, but are available in NVSL-SOP-0068. Researchers seeking additional information on the rRT-PCR method are encouraged to contact NVSL directly.

Extracted RNA samples and reagents were kept on ice for the duration of the reaction setup. In a 96-well plate kept on an ice block, 17 µL of master mix and 8 µL of extracted RNA from samples or controls, was combined. NEC, PEC, PAC, IPC, and NTC controls, as described above, were included and all samples and controls were run in duplicate wells. Once the run was completed, the Ct threshold was adjusted based on the 5% control-based threshold rule by taking the average ΔRn values of the PAC wells at cycle 40 and multiplying it by 0.05, allowing for a consistent baseline measure across all rRT-PCR reactions. Remaining RNA samples were stored at −20 °C for later RT-dPCR analysis.

### RT-dPCR

To create a primer/probe mix for RT-dPCR, 4 µL of each of the 20 pmol/µL primers, 4 µL of the 6 pmol/µL probe, and 4 µL of nuclease-free water was combined in an amber tube and mixed via pulse vortex. The primer/probe mix was combined with Absolute Q 1-Step RT-dPCR Master Mix (4x) and sample RNA to create a reaction mix to be pipetted into the 16-well microfluidic array (MAP16) plate. Extracted RNA samples and reagents were kept on ice for the duration of the reaction setup. Sample reactions were set up and analysis was performed according to Thermo Fisher’s Absolute Q 1-Step RT-dPCR Master Mix (4x) User Guide. No dilution of milk sample RNA was needed as all fell within or below the Absolute Q’s range of detection (~ 1–4.0 × 10^3^ GCs/µL). However, to get the H5N1 stock in range to be precisely quantified, serial dilutions were performed post-extraction, and included a 1/100, 1/200 and 1/500 dilution. Each RT-dPCR plate included an NTC well to establish the baseline false positive ratio, if any.

The input reaction mix is partitioned into 20,480 microchambers where dPCR is performed. The concentration in GCs/µL is determined by the system using Poisson statistics-based calculations from the ratio of negative accepted microchambers to the total number of positive accepted microchambers, the volume of the individual microchambers (432 pL), and any dilution factor (DF) the user inputs into the system. From the concentration reported by the system, the GCs in the original RNA sample were calculated as shown below:

$$ \begin{aligned} \begin{array}{*{20}c} {{\mathrm{GCs}}/\mu {\mathrm{L~in~extracted}}} \\ {{\mathrm{RNA~sample}}} \\ \end{array} {\mathrm{~}} = {\mathrm{~}} & {\mathrm{~}}\frac{{{\mathrm{concentration~}}\left( {{\mathrm{GCs}}/\mu {\mathrm{L}}} \right){\mathrm{~*~~reaction~mix~volume~}}\left( {10{\mathrm{~}}\mu {\mathrm{L}}} \right)}}{{{\mathrm{volume~of~RNA~input~}}\left( {5{\text{ - }}7{\mathrm{~}}\mu {\mathrm{L}}} \right)}} \\ ~ & *~\begin{array}{*{20}c} {{\mathrm{~DF~}}({\mathrm{if~any~and~not~already}}} \\ {{\mathrm{~accounted~for~in~software}})} \\ \end{array} \\ \end{aligned} $$

### Sample Set Up

A set of 16 samples per timepoint was tested at 0, 7, 14, 21, and 28 days post-spiking to assess sample HG/SB. Day 0 samples were extracted on the day of spiking and all other samples were stored at −80 °C for later testing. Samples used for both the HG/SB timepoints and the level of detection studies are outlined in Table 1.

**Table 1:** Target and expected (calculated) genome copies and replicate samples

Target GCs/µL in spiked sample	Expected GCs/µL in extracted sample	Expected GCs/8 µL rRT-PCR reaction input	Number of replicate milk samples per HG/SB timepoint*	Number of replicate milk or PBS samples for LOD calculation*
1.69 × 10^3^	1.25 × 10^3^	1.00 × 10^4^	2	–
3.38 × 10^2^	2.50 × 10^2^	2.00 × 10^3^	–	2
1.69 × 10^2^	1.25 × 10^2^	1.00 × 10^3^	2	–
1.69 × 10^1^	1.25 × 10^1^	1.00 × 10^2^	5	2
1.69	1.25	1.00 × 10^1^	3	–
0.84	0.625	5	2	3
0.68	0.5	4	–	4
0.51	0.375	3	–	4
0.34	0.25	2	–	5
0.17	0.125	1	–	10
Blank	Blank	0	2	1

*Each replicate sample was run in duplicate PCR wells. Statistical analysis considers outcomes from each duplicate rRT-PCR well

### Statistical Evaluation

Data evaluation was performed using a dedicated toolbox of harmonized statistical methods for characterizing samples based on real-time PCR data (QuoData certified). The toolbox is based on internationally standardized statistical approaches defined in relevant ISO standards and widely used in method validation, proficiency testing, and reference material characterization.

In addition, the toolbox includes advanced methodological developments provided by QuoData. These methods are grounded in established statistical principles, are validated in practice, and are essential for assessing method performance, particularly the robustness of assigned values (Uhlig et al., 2015, 2018).

Probabilities of detection (PODs), from which LOD_50%_ values were derived, were modelled using a complementary log-log (cloglog) regression model as recommended in ISO 16140:^2020^, applied in extended form in ISO/TS 27878:^2023^, and implemented in the software µKPI (QuoData GmbH, v0.8.1, 2024). Theoretical details of the approach are described by Uhlig et al. (2015); the AOAC International (2023) POD approach was not applied as it was considered unsuitable for this study.

The degree of the sample heterogeneity was assessed using a random effects model in accordance with ISO 13528:^2022^, allowing separation of within-sample and between-sample variance components (implemented in the software PROLab; QuoData GmbH, v2025.11.6.0,^ 2025^). Additionally, a power-analysis-based procedure, developed by QuoData, was used to determine the minimum level of sample heterogeneity that can be reliably detected given the experimental design and measurement variability, supporting objective interpretation of results on homogeneity testing.

For stability assessment, potential temporal or storage-related effects on measured GC concentrations were considered. Although not yet standardized, this advanced method provides key information for assessing sample stability over time. Specifically, mean GC numbers were estimated using the MU-Hampel procedure, which provides a robust, uncertainty-weighted mean by combining the Hampel estimator with uncertainty propagation, in line with the guide to the expression of uncertainty in measurement (GUM) (JCGM 100:^2008^). This method is described in Uhlig et al. (2018) and implemented as a QuoData-certified procedure.

## Results

### Virus Stability in Milk Samples

**Fig. 1:**
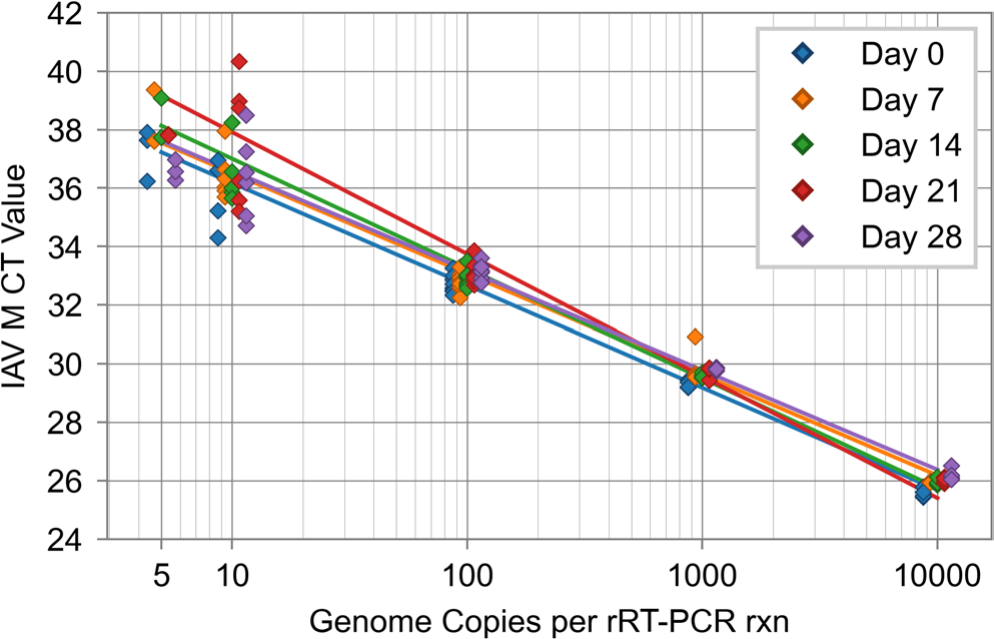
GCs per rRT-PCR Reaction Well and IAV M Ct Values Produced Across HG/SB Timepoints. Diamonds represent the Ct values obtained for each sample’s tested duplicate wells. A linear regression line is included for each timepoint (day)

Virus-spiked milk samples were shown to be stable for 28 days when stored at −80 °C. No significant variation in Ct values was observed over time. Amplification efficiency was high for all days except day 21, ranging from 85% to 97% (Fig. 1). On day 21, amplification efficiency was 74%. No significant differences between timepoints were found (Fig. 1). There was little variability for Ct values across timepoints at 10^2^, 10^3^, and 10^4^ GCs per rRT-PCR reaction well. As expected at lower GCs, the Ct values produced from samples at 10^1^ and 5 GCs had higher variability. Occasional non-detections of samples at 5 and 10^1^ GCs and a well failure from one 10^2^ GC sample resulted in fewer data points than expected (Table 2). Overall, MU-Hampel based average Ct values obtained for each GC level and average Ct differences between GC levels are shown in Table 2.

**Table 2:** Average Ct values produced and Ct value differences

Expected milk sample GCs/rRT-PCR rxn	Average IAV M Ct value for all HG/SB timepoints combined	Average cycle/Ct value difference between GC levels (ΔCt)	Sample size (sample replicates and their duplicate PCR wells, *n*)
10^4^	25.94	–	20
10^3^	29.67	3.73	20
10^2^	32.93	3.26	49
10^1^	36.50	3.57	27
5	37.71	1.21	12

**Fig. 2:**
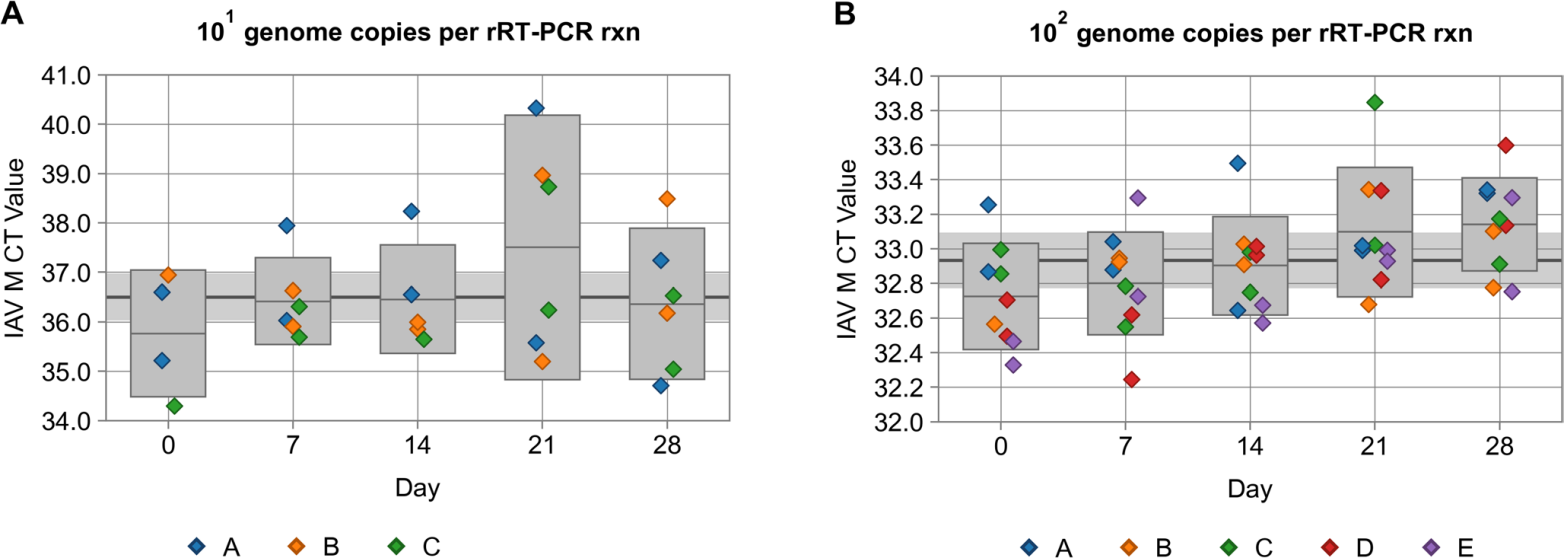
**A** Ct variation of 10^1^ GC samples across timepoints and **B** Ct variation of 10^2^ GC samples across timepoints. Colored diamonds represent 3 distinct sample replicates and their duplicate PCR wells. The overall mean Ct and its 95% confidence interval for all timepoints is shown by the solid black line and its surrounding shaded region spanning the plot. The mean Ct for each day is shown by the thinner black line in the middle of each box

The Ct variation of the two GC sample types with the most data points, 10^1^ GCs and 10^2^ GCs, is outlined further in Fig. 2A and B. 10^1^ GC samples showed more variability in Ct values (Fig. 2A), while 10^2^ GC samples were more consistent (Fig. 2B). Samples at 10^2^ GCs have an overall Ct value average of 32.93, with a minimum value of 32.24 and a maximum value of 33.85. Samples at 10^1^ GCs produced an overall Ct value average of 36.56, with individual Ct values measuring anywhere from 34.29 to 40.33, as well as some non-detections.

### Sample Homogeneity

Based on the Ct values, the between-sample standard deviation (i.e. the S.D. between distinct sample replicates) – as a measure of sample heterogeneity according to ISO 13528:^2022^ – was calculated separately for each GC level and ranges from 0 to 0.10 Ct values, depending on the level (Table 3). These values have already been adjusted for both the day effect and the within-sample standard deviation (i.e. the mean S.D. of duplicates across all distinct sample replicates). In terms of the GCs per rRT-PCR reaction well, this corresponds to a relative between-sample standard deviation ranging from 0.0% to 6.4%.

Since the between-sample standard deviations can only be estimated indirectly and is associated with considerable statistical uncertainty, the unknown theoretical standard deviation at which the estimated between sample standard deviation will be non-zero with a probability of 95% is also specified. According to ISO 11843, this threshold can be interpreted as the minimum detectable value for the level of heterogeneity between samples.

For all five levels, the threshold at which heterogeneity between samples must be assumed is clearly higher than the corresponding calculated standard deviation. Hence, the rRT-PCR results demonstrate a high degree of sample homogeneity.

**Table 3:** Homogeneity of samples

GCs per rRT-PCR rxn	Results for Ct values				Results transformed to the number of genome copies per rRT-PCR rxn		
	Mean ± U (k = 2)	Within-sample s.d.	Between-sample s.d.*	Theoretical s.d. at which between- sample s.d. is non-zero with 95% probability	Within-sample s.d.	Between-sample s.d. *	Theoretical s.d. at which between- sample s.d. is non-zero with 95% probability
5	37.71 ± 0.52	1.00	0.00	1.93	65.5%	0.0%	126.9%
10^1^	36.50 ± 0.47	1.66	0.00	2.25	109.1%	0.0%	147.7%
10^2^	32.93 ± 0.16	0.28	0.10	0.26	18.1%	6.4%	17.2%
10^3^	29.67 ± 0.18	0.33	0.00	0.62	21.5%	0.0%	40.4%
10^4^	25.94 ± 0.20	0.12	0.09	0.22	7.6%	5.7%	14.3%

*The between-sample standard deviation (S.D.) was adjusted for the day effect and for the within-sample S.D

### LOD_50%_ of the rRT-PCR Assay

**Fig. 3:**
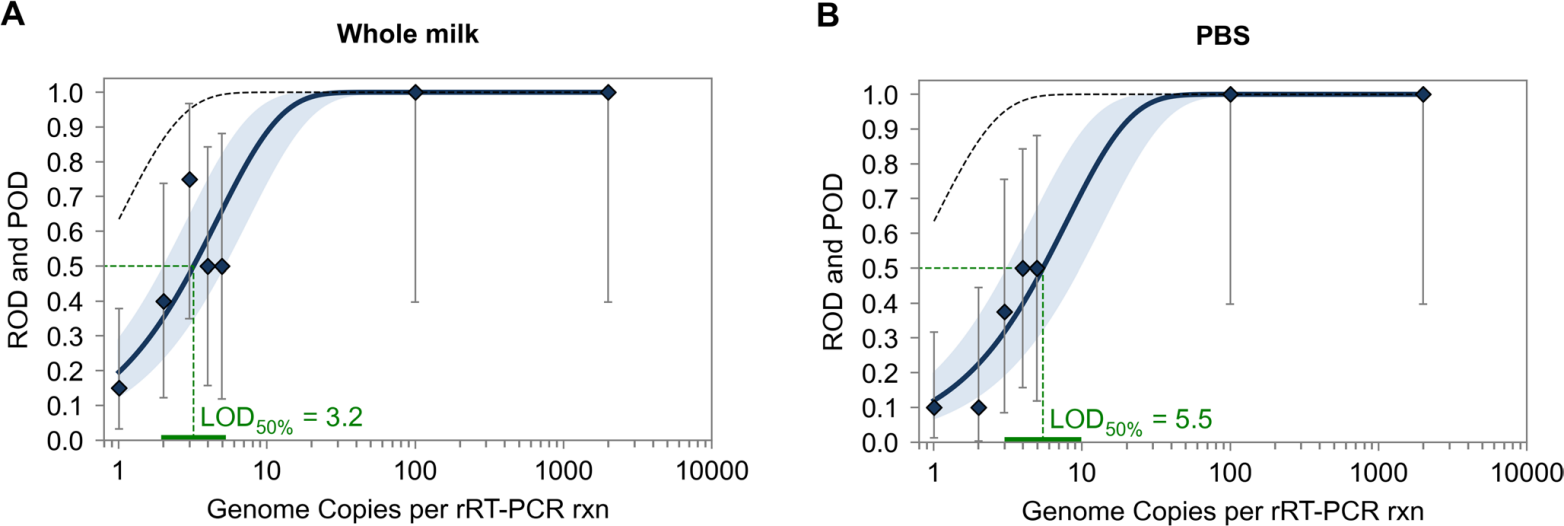
**A** LOD_50%_ of the rRT-PCR Assay obtained from Whole Milk Samples and **B** LOD_50%_ of the rRT-PCR Assay obtained from PBS Samples. LOD_50%_ is shown in green, with the green bar along the x-axis representing its 95% confidence interval. POD is modeled by the solid line, with its associated 95% confidence interval shown by the shaded band. The optimal theoretical POD curve is represented by the dotted line. Diamonds represent the ROD calculated from representative samples tested and error bars show their theoretical 95% confidence interval

The LOD where 50% of the samples are expected to be positive (LOD_50%_), was calculated to be 3.2 GCs per rRT-PCR reaction well for milk samples (Fig. 3A) and 5.5 GCs per rRT-PCR reaction well for PBS samples (Fig. 3B). Little to no detection was observed at 1 GC and detection was variable at 2 to 5 GCs. Rates of detection (RODs) were calculated from the detection of low GC samples tested in duplicate PCR wells. POD was modeled and represented by the solid line in Fig. 3A and B. The optimal POD curve, calculated based on the Poisson model, assumes that the average amplification probability is unaffected by dilution level. 95% confidence intervals of RODs are based on the binomial distribution, which is the appropriate probability model for binary (positive/negative) outcomes of independent replicate rRT-PCR reactions.

### RT-dPCR Confirmation of Milk Sample Genome Copies

**Fig. 4:**
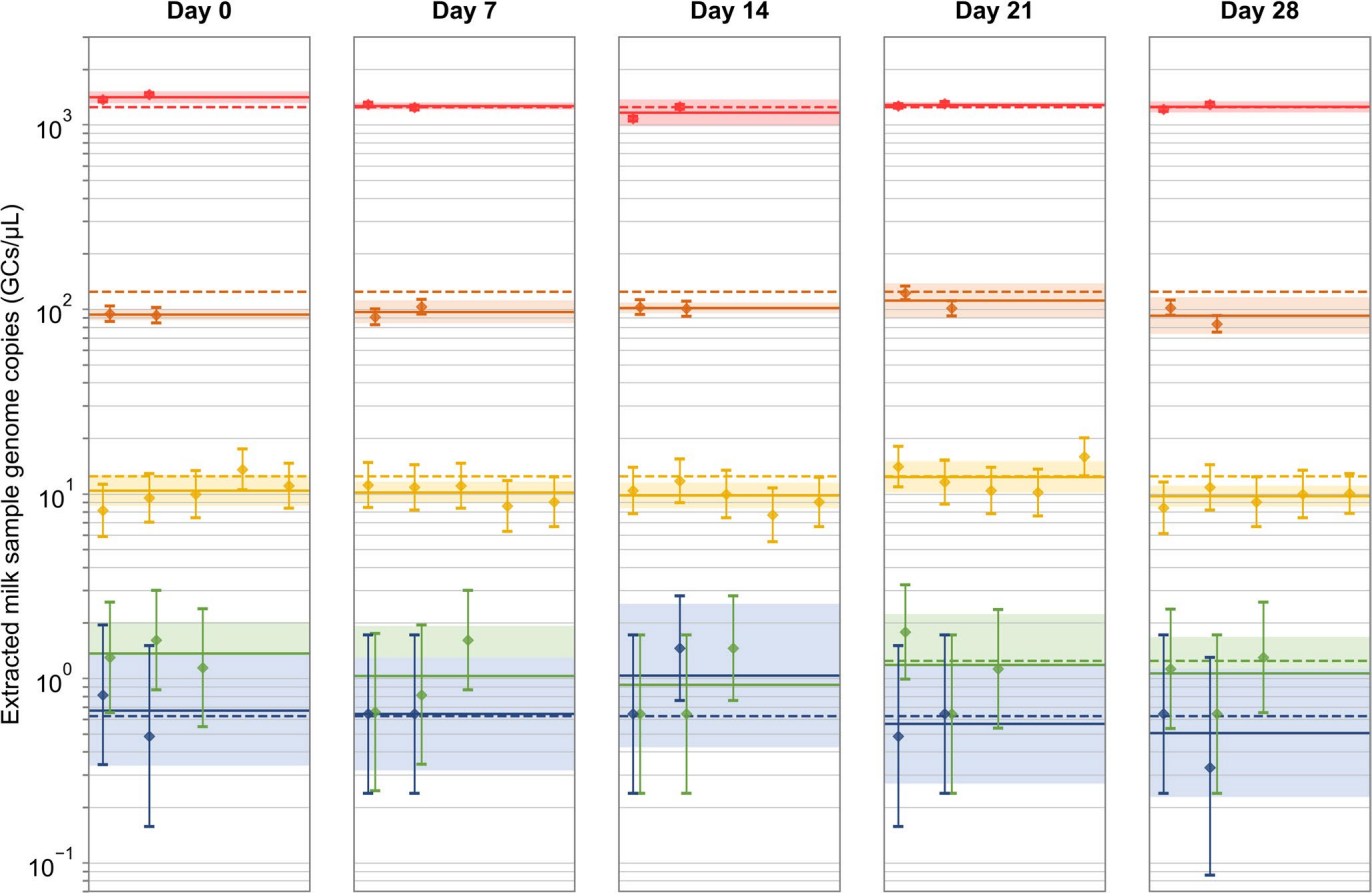
RT-dPCR Results for HG/SB Milk Samples. Colors represent the expected GC copy targets in extracted samples, with 1.25 × 10^3^ GCs/µL in red, 1.25 × 10^2^ GCs/µL in orange, 1.25 × 10^1^ GCs/µL in yellow, 1.25 GCs/µL in green and 0.625 GCs/µL in blue. Solid lines and shaded bands represent the calculated mean value with the 95% confidence intervals. Dashed lines represent the calculated expected values. Diamonds and whiskers show the measured dPCR values of milk samples and their 95% confidence intervals obtained from the Absolute Q analysis

The measured GCs/µL of extracted milk samples obtained from RT-dPCR aligned closely with the calculated expected values (as referenced in Table 1). Moreover, samples of the same replicate level show consistent GCs across timepoints (Fig. 4). No day-specific trends or instabilities were observed in the measured GCs or their variability. As expected, and as previously seen in rRT-PCR, variability is higher for samples with low expected GCs, 1.25 GCs/µL and 0.625 GCs/µL. Samples at 1.25 × 10^1^ expected GCs/µL and 1.25 × 10^2^ GCs/µL measured lower than calculated and samples expected at 1.25 × 10^3^ GCs/µL were consistent with the calculated value and are both accurate and precise. The overall averages of the measured extracted sample RNA GCs are as follows, expressed in GCs/µL: 1.28 × 10^3^, 9.95 × 10^1^, 1.05 × 10^1^, 1.10, and 0.679.

### Discussion

This study was a collaborative effort between Vet-LIRN and the MPTL to evaluate a rRT-PCR method based on NVSL-SOP-0068 and NVSL-WI-1843 for the detection of HPAI in milk, to ultimately support the development of future interlaboratory exercises. Work described here shows that MPTL can reliably produce stable, precise, and homogenous samples which can be used for future nationwide PEs.

### Homogeneity and Stability of Samples

Because laboratories may use different methodologies, PE samples received by each participant should contain the same target organism demonstrating equivalent established characteristics; providers are required to confirm this, along with sample HG/SB according to ISO 22117:^2019^ and ISO 17043:^2023^. Proper HG/SB ensures samples produce intended results and is the core parameter of all PEs, as it is critical for ensuring consistency across replicate samples prepared for a PE and their reliability in downstream analyses.

Quantitative evaluation of HG/SB requires quantitative measurement techniques. Accordingly, the assessments in this study were based on Ct values produced from the NVSL’s IAV M rRT-PCR assay, converted into estimated GC numbers. Despite the inherent limitations in the precision of Ct measurements, the results demonstrated both high homogeneity and high stability of the samples (Fig. 1; Table 3). To obtain a more accurate determination of the true GC numbers, the evaluation was supplemented by RT-dPCR measurements of the extracted samples, providing a more reliable quantification and confirming the sample GC numbers (Fig. 4). No significant trends were observed between timepoints, indicating sample stability for at least 28 days. This stability of samples provides an extended window, allowing PE providers to test final samples for HG prior to shipment, with ample time still leftover for participating laboratories to begin testing of samples and still obtain the expected results.

The precision of the spiked samples should also be considered. Not only are the samples described here homogeneous and stable, but the method used to deliver intended GC numbers in spiked samples is also precise. According to Spackman (2020), the originator of the IAV M rRT-PCR method, there is an expected cycle difference of 3–4 cycles between samples that have a tenfold difference in starting RNA, which was consistently obtained in rRT-PCR data (Table 2). This pattern is consistent between rRT-PCR and RT-dPCR. For example, via RT-dPCR samples expected at 1.25 × 10^2^ GCs/µL measured at 9.95 × 10^1^ GCs/µL and samples expected at 1.25 × 10^1^ GCs/µL measured at 1.05 × 10^1^ GCs/µL; this near-perfect tenfold difference is also shown in the rRT-PCR, where the difference in the average Ct values obtained between samples at these two levels was 3.26, indicative of this tenfold difference with near-perfect reaction efficiency (Spackman, 2020).

### Sensitivity of the NVSL’s IAV M rRT-PCR Assay for HPAI Detection in Milk Samples

Through the evaluation of the samples as described above, we can also characterize the performance of this PCR assay specifically for the detection of HPAI in milk samples, which has not yet been publicly evaluated. However, a potential limitation of this work is the use of pasteurized store-bought milk to simulate samples. Though pasteurized milk is a good standard matrix for PE samples because retail preparation limits possible matrix-related effects, differences may exist between real-world bulk tank milk samples commonly used for HPAI screening and the simulated milk samples used in this study.

As the limits of a rRT-PCR assay’s detection are approached, extraction efficiency becomes key in detection. Therefore, in this case, using a kit that is validated for milk matrices, like the MagMAX CORE, is very important for efficient isolation of genetic material from the sample. For low GC samples, distribution of the genetic material in extracts also becomes more random, following a Poisson-like distribution, resulting in a mix of positive and negative results and thus allowing for the calculation of the LOD_50%_ (Uhlig et al., 2015). Therefore, establishing the LOD_50%_ allows PE providers to effectively challenge participants, but including clear positive samples is also crucial. Distributing samples with a step-down approach, from highly positive samples to samples at or near the LOD_50%_, results in rich datasets; the analysis of which allows participating laboratories to confirm the limitations of their procedures, i.e. at which level does their method start to become unreliable?

The LOD_50%_ for milk, (3.2 GCs/rRT-PCR rxn), was lower than PBS (5.5 GCs/rRT-PCR rxn). However, it must be noted that the data point for samples at 3 GCs was produced from only 4 replicate samples (8 PCR wells). For milk samples, this resulted in higher positivity than expected, falling above the POD curve. If more replicates are tested, the 3 GC data point is likely to fall more in line with the POD curve, aligning the obtained LOD_50%_ of milk samples closer to that of PBS samples.

The difference in LOD_50%_ for milk and PBS was also not significant, indicating that, in this study, milk did not have a matrix effect (Fig. 3A & B). Although data was not shown, both a PEC and IPC were included to identify matrix inhibition, and no inhibitory effect was observed for both PBS and milk samples. This indicates minimal PCR inhibition from milk, aligning with reports that inhibition in milk largely stems from calcium or milk proteins and can be mitigated by optimized extraction techniques (Bickley et al., 1996). In this case, dilution of the milk sample prior to extraction with the MagMAX CORE kit proved to be a reliable way to minimize matrix inhibition.

### Analysis and Special Considerations for Low GC Samples

The Absolute Q dPCR system is known to be more precise at ranges of ~ 4.50 × 10^3^ – 4.50 × 10^4^ GCs/µL of sample but has high sensitivity and can detect 1 GC in the entire array/well reaction (ThermoFisher Scientific, n.d.). However, it still relies on the Poisson distribution to determine sample GCs. Owing to this lower precision, 95% confidence intervals may become larger at lower GCs, but as demonstrated here, the Absolute Q still differentiated between different GC milk samples with high consistency and produced GC values that closely agree with expected values in samples (Table 1; Fig. 4). As discussed previously, the consistent measurements obtained in RT-dPCR results (Fig. 4) affirm the results of the rRT-PCR analysis and prove that the back-calculation and dilution method use to spike samples at intended levels is relatively accurate and precise.

As GCs in samples approach the LOD_50%_, variation in detection, Ct value, and copy number determination increases. This variability observed at lower GC levels is a known effect of the Poisson distribution, and may be due to both measurement variability of the RT-PCR assays themselves and sampling inconsistencies; the lower the amount of genetic material in the extracted sample, the higher the likelihood that the volume input into rRT-PCR does not actually contain the target (Ki et al., 2021; Ellison et al., 2006). Of course, sample handling, extraction efficiency, and storage conditions should also be considered, as genetic material could be lost or degraded throughout the process.

In most traditional PT schemes, fractional results produced from low GC samples, like the ones described here, would be disregarded. Low GC samples were intentionally included in this study to evaluate the sensitivity and reliability of the NVSL’s IAV M rRT-PCR method in MTPL hands. Detection of low GC samples also has implications for the early detection of HPAI in milk. A previous study found the LOD to be 10^4^ copies/mL (10^1^ GCs/µL), which translates to 1 infected cow in a herd of 100 shedding 10^6^ H5N1 copies/mL (Stachler et al., 2025). Identifying the virus at low GCs can allow laboratories to detect infections during the initial stages, but the sensitivity of current methodology was previously not well defined (Stenkamp-Strah et al., 2025). Even when viral loads may be minimal, transmission risk between cows remains high. Bulk tank milk sampling was found to be sensitive for early outbreak detection, but frequent sampling is needed and is essential not only for containment measures, but also for preventing the further spread of HPAI within and between dairy herds, as well as minimizing potential cross-species transmission (Le Sage et al., 2024; Morse et al., 2024; Stenkamp-Stram et al., 2025).

### Overall Conclusions

This study reports on the production of high-quality proficiency samples, allowing for a fit-for-purpose evaluation of the NVSL’s IAV M rRT-PCR method for detection of HPAI A (H5N1) in milk. Using inactivated H5N1 spiked into milk over a series of GC levels, we successfully confirmed: (i) virus stability in milk samples stored at − 80 °C; (ii) sample HG/SB for 28 days across spike levels; (iii) evaluation of sample agreement shown using rRT-PCR and RT-dPCR-to assess GC values and (iv) method sensitivity, using POD as a function of GC level in milk versus in PBS to determine the LOD_50%_. Importantly, these elements all play a key role in PE development.

Overall, our results confirm that the implemented method is suitable for verification of PE samples and that the sample-preparation approach used to generate spiked milk items can produce PE materials that are sufficiently homogeneous and stable for use in future interlaboratory exercises. The presented data provide a practical framework for future HPAI milk PE programs and support the use of this NVSL-based method as an internal characterization assay for verifying PE sample quality.

## Data Availability

The datasets generated during the current study are available from the corresponding author upon request.

## References

[CR1] AOAC International (2023). Appendix J: AOAC International methods committee guidelines for validation of microbiological methods for food and environmental surfaces. In G. W. Latimer, Jr. (Ed.), *Official Methods of Analysis of AOAC International* (22nd ed.).

[CR2] Bickley, J. K., Short, D. G., McDowell, & Parkes, H. C. (1996). Polymerase chain reaction (PCR) detection of *Listeria monocytogenes* in diluted milk and reversal of PCR inhibition caused by calcium ions. *Letters in Applied Microbiology*, *22*, 153–158. 10.1111/j.1472-765X.1996.tb01131.x 8936376 10.1111/j.1472-765x.1996.tb01131.x

[CR3] Burrough, E. R., Magstadt, D. R., Petersen, B., Timmermans, S. J., Gauger, P. C., Zhang, J., Siepker, C., Mainenti, M., Li, G., Thompson, A. C., Gorden, P. J., Plummer, P. J., & Main, R. (2024). Highly pathogenic avian influenza A(H5N1) clade 2.3.4.4b virus infection in domestic dairy cattle and cats, United States, 2024. *Emerging Infectious Diseases,**30*(7), 1335–1343. 10.3201/eid3007.240508 38683888 10.3201/eid3007.240508 PMC11210653

[CR4] Caserta, L. C., Frye, E. A., Butt, S. L., Laverack, M., Nooruzzaman, M., Covaleda, L. M., Thompson, A. C., Koscielny, M. P., Cronk, B., Johnson, A., Kleinhenz, K., Edwards, E. E., Gomez, G., Hitchener, G., Martins, M., Kapczynski, D. R., Suarez, D. L., Alexander Morris, E. R., Hensley, T., & Diel, D. G. (2024). Spillover of highly pathogenic avian influenza H5N1 virus to dairy cattle. *Nature,**634*(8034), 669–676. 10.1038/s41586-024-07849-4 39053575 10.1038/s41586-024-07849-4 PMC11485258

[CR5] Deng, K., Nemser, S. M., Frost, K., Goodman, L. B., Ip, H. S., Killian, M. L., Ulaszek, J., Kiener, S., Kmet, M., Uhlig, S., Hettwer, K., Colson, B., Nichani, K., Schlierf, A., Tkachenko, A., Miller, M. R., Reddy, R., & Tyson, G. H. (2023). Successful detection of delta and omicron variants of SARS-CoV-2 by veterinary diagnostic laboratory participants in an interlaboratory comparison exercise. *The Journal of Applied Laboratory Medicine*, *8*(4), 726–741. 10.1093/jalm/jfad018 37222567 10.1093/jalm/jfad018 PMC11555767

[CR6] Ellison, S. L., English, C. A., Burns, M. J., & Keer, J. T. (2006). Routes to improving the reliability of low level DNA analysis using real-time PCR. *BMC Biotechnology,**6*, Article 33. 10.1186/1472-6750-6-33 16824215 10.1186/1472-6750-6-33 PMC1559608

[CR7] ISO 13528:2022. Statistical methods for use in proficiency testing by interlaboratory comparison, August 2022.

[CR35] ISO 16140-4:2020. Microbiology of the food chain - Method validation Part 4: Protocol for method validation in a single laboratory, July 2020.

[CR9] ISO 17043:2023. Conformity assessment – General requirements for the competence of proficiency testing providers, May 2023.

[CR10] ISO 22117:2019. Microbiology of the food chain – Specific requirements and guidance for proficiency testing by interlaboratory comparison, February 2019.

[CR11] ISO/TS 27878:2023. Reproducibility of the level of detection (LOD) of binary methods in collaborative and in-house validation studies, January 2023.

[CR12] JCGM 100:2008. (Gum 1995 with minor corrections). Evaluation of measurement data – Guide to the expression of uncertainty in measurement, September 2008.

[CR13] Ki, U., Suzuki, T., Nakazawa, S., Yonekawa, Y., Wantanabe, K., Hashimoto, M., Hatada, S., & Unno, H. (2021). Evaluation method for asymmetric uncertainty of quantitative polymerase chain reaction measurements of deoxyribonucleic acids with low copy number. *Scientific Reports,**11*, Article 11550. 10.1038/s41598-021-90959-0 34078977 10.1038/s41598-021-90959-0 PMC8172552

[CR14] Le Sage, V., Campbell, A. J., Reed, D. S., Duprex, W. P., & Lakdawala, S. S. (2024). Persistence of influenza H5N1 and H1N1 viruses in unpasteurized milk on milking unit surfaces. *Emerging Infectious Diseases,**30*(8), 1721–1723. 10.3201/eid3008.240775 38914418 10.3201/eid3008.240775 PMC11286056

[CR15] Morse, J., Coyle, J., Mikesell, L., Stoddard, B., Eckel, S., Weinberg, M., Kuo, J., Riner, D., Margulieux, K., Stricklen, J., Dover, M., Kniss, K. L., Jang, Y., Kirby, M. K., Frederick, J. C., Lacek, K. A., Davis, C. T., Uyeki, T. M., Lyon-Callo, S., & Bagdasarian, N. (2024). Influenza A (H5N1) virus infection in two dairy farm workers in Michigan. *New England Journal of Medicine*, *391*(10), 963–964. 10.1056/NEJMc2407264 39115081 10.1056/NEJMc2407264

[CR16] Mostafa, A., Naguib, M. M., Nogales, A., Barre, R. S., Stewart, J. P., García-Sastre, A., & Martinez-Sobrido, L. (2024). Avian influenza A (H5N1) virus in dairy cattle: Origin, evolution, and cross-species transmission. *mBio*, *15*(12), e02542–e02524. 10.1128/mbio.02542-24 39535188 10.1128/mbio.02542-24 PMC11633217

[CR17] National Animal Health Laboratory Network (NVSL). (2023). Real-time RT-PCR Detection of Influenza A and Avian Paramyxovirus type-1 (NVSL-SOP-0068.05). https://www.aphis.usda.gov/labs/nahln/approved-labs/nahln-document-list

[CR18] National Animal Health Laboratory Network (NVSL). (2024). NVSL Guidance for Molecular Testing of Milk Samples for Influenza A (NVSL-WI-1843). https://www.aphis.usda.gov/labs/nahln/approved-labs/nahln-document-list

[CR19] Nemser, S., Lindemann, S., Chen, Y., Lopez, S., Pickens, S., Ulaszek, J., Kmet, M., Powers, C., Ensley, S., Schrunk, D., Rumbeiha, W., Tkachenko, A., Guag, J., Ceric, O., Jones, J., Reimschuessel, R., & Reddy, R. (2021). A review of proficiency exercises offered by the Veterinary Laboratory Investigation and Response Network (Vet-LIRN) and Moffett Proficiency Testing Laboratory from 2012 to 2018. *Accreditation and Quality Assurance,**26*, 143–156. 10.1007/s00769-021-01471-x

[CR20] QuoData GmbH. (2025). *PROLab Plus:** Software for interlaboratory comparisons* (Version 2025.11.6.0). https://quodata.de/de/prolab

[CR21] QuoData GmbH. (n.d.). *QuoData Certified*. Accessed on 02/05/2026. https://quodata.de/de/quodata-certified

[CR34] QuoData GmbH. (2024). *µKPI: Soluti​ons for quality assurance and data quality - Suite of tools for microbiological method key performance indicators.* https://microkpi.quodata.de/

[CR22] Singh, N., Miller, M. R., Nemser, S. M., Tkachenko, A., Uhlig, S., Frost, K., Hettwer, K., Ulaszek, J., Kmet, M., Wang, L., Allender, M. C., & Reddy, R. (2025). Proficiency test of SARS-CoV-2 Omicron variant detection in diagnostics samples by veterinary diagnostic laboratories. *Accreditation and Quality Assurance,**30*, 45–53. 10.1007/s00769-024-01622-w

[CR23] Spackman, E. (2020). Avian influenza detection and quantitation by real-time RT-PCR. *Methods in Molecular Biology*. 10.1007/978-1-0716-0346-8_11. 2123. 32170686 10.1007/978-1-0716-0346-8_11

[CR24] Spackman, E., & Lee, S. A. (2020). Avian influenza virus RNA extraction. *Methods in Molecular Biology*. 10.1007/978-1-0716-0346-8_10. 2123. 32170685 10.1007/978-1-0716-0346-8_10

[CR26] Stachler, E., Gnirke, A., McMahon, K., Gomez, M., Stenson, L., Guevara-Reyes, C., Knoll, H., Hill, T., Hill, S., Messer, K. S., Arizti-Sanz, J., Albeez, F., Curtis, E., Samani, P., Wewior, N., O’Connor, D. H., et al. (2025). Establishing methods to monitor influenza (A)H5N1 virus in dairy cattle milk, Massachusetts, USA. *Emerging Infectious Diseases,**31*(13), 70–75. 10.3201/eid3113.250087 40138725 10.3201/eid3113.250087 PMC12078538

[CR27] Stenkamp-Stram, C., Lombard, J., Melody, B., Brinson, P., & McCluskey, B. (2025). Influenza A virus detection in bulk tank milk from dairies affected by highly pathogenic avian influenza H5N1. *medRxiv*. 10.1101/2025.10.26.25338833

[CR28] ThermoFisher Scientific. (n.d.).*QuantStudio Absolute Q Digital PCR System, desktop* – *FAQs.* Accessed on 02/05/2026. https://www.thermofisher.com/order/catalog/product/A52864/faqs

[CR29] Uhlig, S., Colson, B., & Gowik, P. (2018). Taking laboratory uncertainties into account in the Hampel estimator. *Accreditation and Quality Assurance,**24*(1), 25–32. 10.1007/s00769-018-1332-x

[CR30] Uhlig, S., Frost, K., Colson, B., Simon, K., Mäde, D., Reiting, R., Gowik, P., & Grohmann, L. (2015). Validation of qualitative PCR methods on the basis of mathematical–statistical modelling of the probability of detection. *Accreditation and Quality Assurance*, *20*, 75–83. 10.1007/s00769-015-1112-9

[CR31] USDA. *Actions to Protect Livestock Health From Highly Pathogenic H5N1 Avian Influenza | Home*. (2024, April 24). https://www.usda.gov/about-usda/news/press-releases/2024/04/24/usda-actions-protect-livestock-health-highly-pathogenic-h5n1-avian-influenza. Accessed on 12/08/2025.

[CR32] USDA. *NAHLN Document List* (2025, December 29). https://www.aphis.usda.gov/labs/nahln/approved-labs/nahln-document-list. Accessed on 01/28/2026.

[CR33] USDA. *National Milk Testing Strategy* (2025, November 17). https://www.aphis.usda.gov/livestock-poultry-disease/avian/avian-influenza/hpai-detections/livestock/nmts. Accessed on 12/08/2025.

